# Classification of postoperative pancreatic fistula after left pancreatectomy: international multicentre cohort study

**DOI:** 10.1093/bjsopen/zraf149

**Published:** 2025-12-03

**Authors:** Akseli Bonsdorff, William Yu, Jakob Kirkegård, Charles de Ponthaud, Trond Kjeseth, Poya Ghorbani, Johanna Wennerblom, Caroline Williamsson, Alexandra W Acher, Manoj Thillai, Timo Tarvainen, Aki Uutela, Jukka Sirén, Arto Kokkola, Dyre Kleive, Mushegh Sahakyan, Rolf E Hagen, Andrea Lund, Mette Fugleberg Nielsen, Richard Fristedt, Christina Biörserud, Svein Olav Bratlie, Bobby Tingstedt, Knut J Labori, Sébastien Gaujoux, Stephen J Wigmore, Julie Hallet, Ernesto Sparrelid, Ville Sallinen

**Affiliations:** Department of Gastroenterological Surgery, Helsinki University Hospital and University of Helsinki, Helsinki, Finland; Division of Surgery and Oncology, Department of Clinical Science, Intervention, and Technology, Karolinska Institutet, Karolinska University Hospital, Stockholm, Sweden; Department of Surgery, Aarhus University Hospital, HPB Section and Institute for Clinical Medicine, Aarhus University, Aarhus, Denmark; Department of Digestive, Hepato-Pancreato-Biliary Surgery and Liver Transplantation, AP-HP Pitié-Salpêtrière Hospital, Sorbonne Université Paris, Paris, France; Department of Hepato-Pancreato-Biliary Surgery, Rikshospitalet, Oslo University Hospital, Oslo, Norway; Department of Clinical Medicine, University of Bergen, Bergen, Norway; Division of Surgery and Oncology, Department of Clinical Science, Intervention, and Technology, Karolinska Institutet, Karolinska University Hospital, Stockholm, Sweden; Department of Surgery, Sahlgrenska University Hospital, Gothenburg, Sweden; Department of Surgery, Lund University, Skåne University Hospital at Lund, Lund, Sweden; Department of Surgery, University of Toronto and Sunnybrook Health Sciences Centre, Toronto, Ontario, Canada; Hepatobiliary and Pancreatic Unit & Edinburgh Transplant Unit, University of Edinburgh, Royal Infirmary, Edinburgh, UK; Department of Gastroenterological Surgery, Helsinki University Hospital and University of Helsinki, Helsinki, Finland; Hepatobiliary and Pancreatic Unit & Edinburgh Transplant Unit, University of Edinburgh, Royal Infirmary, Edinburgh, UK; Department of Transplantation and Liver Surgery, Helsinki University Hospital and University of Helsinki, Helsinki, Finland; Department of Gastroenterological Surgery, Helsinki University Hospital and University of Helsinki, Helsinki, Finland; Department of Gastroenterological Surgery, Helsinki University Hospital and University of Helsinki, Helsinki, Finland; Department of Hepato-Pancreato-Biliary Surgery, Rikshospitalet, Oslo University Hospital, Oslo, Norway; Department of Surgery, Vestre Viken Hospital Trust, Ringerike Hospital, Hønefoss, Norway; The Intervention Centre, Oslo University Hospital, Rikshospitalet, Oslo, Norway; Department of Surgery N1, Yerevan State Medical University, Yerevan, Armenia; Department of Surgery, Vestfold Hospital Trust, Tønsberg, Norway; Department of Surgery, Aarhus University Hospital, HPB Section and Institute for Clinical Medicine, Aarhus University, Aarhus, Denmark; Department of Surgery, Aarhus University Hospital, HPB Section and Institute for Clinical Medicine, Aarhus University, Aarhus, Denmark; Department of Surgery, Lund University, Skåne University Hospital at Lund, Lund, Sweden; Department of Surgery, Sahlgrenska University Hospital, Gothenburg, Sweden; Department of Surgery, Sahlgrenska University Hospital, Gothenburg, Sweden; Department of Surgery, Lund University, Skåne University Hospital at Lund, Lund, Sweden; Department of Hepato-Pancreato-Biliary Surgery, Rikshospitalet, Oslo University Hospital, Oslo, Norway; Institute of Clinical Medicine, University of Oslo, Oslo, Norway; Department of Digestive, Hepato-Pancreato-Biliary Surgery and Liver Transplantation, AP-HP Pitié-Salpêtrière Hospital, Sorbonne Université Paris, Paris, France; Hepatobiliary and Pancreatic Unit & Edinburgh Transplant Unit, University of Edinburgh, Royal Infirmary, Edinburgh, UK; Department of Surgery, University of Toronto and Sunnybrook Health Sciences Centre, Toronto, Ontario, Canada; Division of Surgery and Oncology, Department of Clinical Science, Intervention, and Technology, Karolinska Institutet, Karolinska University Hospital, Stockholm, Sweden; Department of Gastroenterological Surgery, Helsinki University Hospital and University of Helsinki, Helsinki, Finland; Department of Transplantation and Liver Surgery, Helsinki University Hospital and University of Helsinki, Helsinki, Finland

## Abstract

**Background:**

Postoperative pancreatic fistula (POPF) is a major complication after left pancreatectomy. The current International Study Group of Pancreatic Surgery classification has limitations, including heterogeneity in morbidity and high interobserver variability. This study aimed to assess POPF-related morbidity after left pancreatectomy and propose a refined classification system.

**Methods:**

Patients undergoing left pancreatectomy at nine high-volume centres between January 2010 and April 2023 were included. All postoperative treatments and interventions related to POPF were collected. The Comprehensive Complication Index (CCI) was used to assess total cumulative morbidity. The International Study Group of Pancreatic Surgery B POPF was subclassified (B1 = prolonged drainage, B2 = pharmacological intervention, B3 = percutaneous intervention, B4 = endoscopic or angiographic intervention). A new POPF grading system was developed by combining subclasses with similar morbidity.

**Results:**

Among 2284 patients, 497 (21.8%) had B (B1: 48 (2.1%), B2: 135 (5.9%), B3: 175 (7.7%), B4: 99 (4.3%)) or C (40 (1.8%)) POPF. Median (interquartile range) POPF-related CCI was 33.5 (22.6–39.7). A significant overlap existed between B and C POPF in terms of CCI. Median CCI (i.q.r.) increased with the B POPF subclasses (B1–B4), 8.7 (8.7-8.7) – 22.6 (20.9-22.6) – 33.5 (33.5-34.6) – 47.4 (39.7-52.1) (*P* < 0.001), but no difference between B4 POPF and C POPF was observed (median CCI 47.4 *versus* 50.2; *P* = 0.265). The refined POPF grading system consists of grades 0 (including biochemical leak and B1), A (including B2), B (including B3), and C (including B4 and C) reflecting worsening morbidity.

**Conclusion:**

The current International Study Group of Pancreatic Surgery classification includes highly heterogeneous grade B POPF cases, ranging from minimal to severe morbidity. The refined grading system improves classification and clinical relevance by aligning POPF severity with morbidity and short-term outcomes.

## Teaser

This multicentre study of 2284 patients proposes a refined classification system for postoperative pancreatic fistula after left pancreatectomy, addressing the heterogeneity of morbidity within the current International Study Group of Pancreatic Surgery definition. By analysing the severity and clinical consequences of different interventions, the study suggests a new three-tiered grading (A, B, C) that better correlates with patient outcomes and improves consistency between centres.

## Introduction

Postoperative pancreatic fistula (POPF) is the main driver for morbidity after left pancreatectomy, with its reported incidence ranging between 5% and 35%, with a median of 20%^1–5^. In the current 2016 International Study Group of Pancreatic Surgery (ISGPS) definition^6^, a fistula of pancreatic fluid requiring prolonged intra-abdominal drainage or postoperative percutaneous intervention, administration of somatostatin analogues or antibiotics, or endoscopic/angiographic procedures is classified as grade B POPF. Grade C POPF is defined as a fistula requiring reoperation, resulting in organ failure, leading to intensive care unit admission or death. Grades B and C are usually combined as clinically relevant POPF, which has been routinely used as a primary outcome in the majority of studies investigating pancreatic fistula^7^. Of note, the grade A fistula no longer exists as the former grade A fistula was renamed to biochemical leak in the 2016 update.

Recent publications have underlined some potential pitfalls with the current POPF definition. In 2019, Maggino *et al*.^8^ noted grade B POPF to include patients with significantly differing consequences. They divided the B grade into three different categories: prolonged drainage only, pharmacological treatments, and interventional procedures. A rising trend in length of hospital stay (LOS), readmissions, costs, and other postoperative adverse events was observed advancing across the subclasses. The rate of interventional procedures was observed to be higher for patients undergoing left pancreatectomy compared with pancreatoduodenectomy; however, more comprehensive analyses for left pancreatectomy only were not presented. In addition, B subclasses were not compared with C POPF. These results were further validated in 2022^9^, albeit only for pancreatoduodenectomy, and prolonged drainage was shown to have clinical and economic consequences similar to biochemical leak. Worryingly, just recently, Hendriks *et al*.^10^ demonstrated the ISGPS-defined complications to have significant interobserver variability, especially between centres, to the extent that improving the definitions in future studies was deemed very important.

The aim of this study was to assess the heterogeneity of POPF-related morbidity and propose a refined and potentially more homogenous classification for clinically relevant POPF after left pancreatectomy.

## Methods

### Participants

Consecutive patients undergoing left pancreatectomy at nine different high-volume pancreatic surgery centres (eight located in Europe, one in North America) between 1 January 2010 and 1 April 2023 were screened for inclusion. Inclusion criteria were age 18 years or over, and open or minimally invasive left pancreatectomy with or without splenectomy. Patients undergoing any type of vascular resection were included but multivisceral resections (other than splenectomy) were excluded. Patient data were obtained using medical records reviewed by each participating centre according to their local regulations and legislation. The study had institutional review board approval from each of the participating centres.

### Data collection

In addition to basic demographics and perioperative variables, each unique postoperative intervention or treatment-targeting POPF was collected for each patient. They were categorized as prolonged drainage (> 21 postoperative days), pharmacological treatment (antibiotics or somatostatin analogues), percutaneous intervention, angiographic intervention, endoscopic intervention, and reoperation.

### Definitions

ISGPS definitions for POPF, postpancreatectomy haemorrhage (PPH), and delayed gastric emptying (DGE) were used^6,11,12^. The Clavien–Dindo classification-based Comprehensive Complication Index (CCI)^13,14^ was used to assess POPF-related cumulative morbidity. It cumulatively considers each intervention and treatment the patient underwent and scores them according to severity. This score is then scaled between 0 and 100, where 0 equals no morbidity and 100 equals mortality. For example, antibiotic treatment (Clavien–Dindo II) followed with percutaneous intervention (Clavien–Dindo IIIa) leads to a CCI of 33.5. Postoperative mortality was defined as occurring within 90 postoperative days.

### Proposed subclassification for POPF

**Fig. 1:**
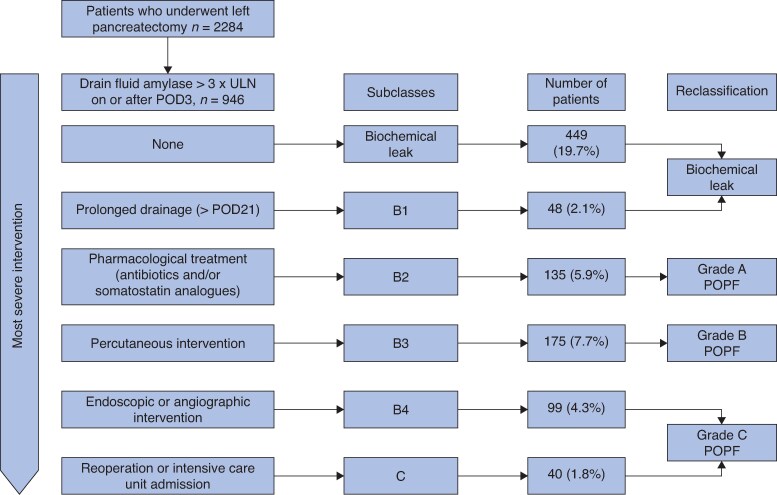
Illustration of the proposed subclassification for postoperative pancreatic fistula after left pancreatectomy and the distribution of these grades in patients who underwent left pancreatectomy ULN, upper limit of normal; POD, postoperative day.

Subclassification was approached by recategorizing the different components of the current ISGPS grade B POPF definition. The interventions for POPF were thought to be hierarchical from the least invasive to the most invasive or severe as follows: prolonged drainage of intraoperatively placed drain (> 21 days) (Clavien–Dindo I)—pharmacological treatment (Clavien–Dindo II)—percutaneous intervention (Clavien–Dindo IIIa)—angiographic/endoscopic intervention (Clavien–Dindo IIIa or IIIb), in line with the proposal from Maggino *et al*^8^. *Figure 1* shows the four-tier subclassification for grade B POPF. The final proposal for the new POPF grading system was developed by omitting subclasses with morbidity comparable to no POPF and by combining subclasses with similar morbidity.

### Statistics

Continuous data are presented as median (interquartile range (i.q.r.)), and categorical data as frequency and percentage. Differences between continuous variables were assessed statistically with the non-parametric Mann–Whitney *U* test for two groups and Kruskal–Wallis test for numerous groups. These tests do not assume normality but do assume independence of observations and similar-shaped distributions across groups for meaningful comparison of medians. Post hoc pairwise testing for continuous data over numerous categories was performed with Dunn’s test with Bonferroni correction to adjust for multiple testing, a total of five comparisons (B1, B2, B3, B4, and C) was made, and adjusted *P*-values were calculated as p_adjusted_ = p_raw_ × 5. For categorical data, differences were assessed statistically using the χ^2^ test. Logistic regression was used to calculate odds ratios (with 95% confidence intervals (c.i.)) for outcomes per POPF grade to demonstrate effect size. Likewise, linear regression was used for continuous variables, and coefficients are reported. A two-sided *P*-value < 0.05 was considered statistically significant. All analyses were performed with SPSS™ v. 28 (IBM, Armonk, NY).

## Results

### Participants

In total, 2284 patients underwent left pancreatectomy during the study period, of which 497 (21.8%) experienced B or C POPF. Basic demographics of the whole cohort are presented in *Table 1*, and perioperative factors per participating centre are reported in *Table S1*.

**Table 1:** Basic demographics of 2284 patients undergoing left pancreatectomy

	Full cohort (*n* = 2284)*
Age (years), median (i.q.r.)	65 (53–72)
**Sex**	
Male	1058 (46.3%)
Female	1226 (53.7%)
BMI (kg/m^2^), median (i.q.r.)	26.0 (23.0–29.0)
History of cardiovascular disease†	205 (9.0%)
History of chronic pulmonary disease	224 (9.8%)
History of diabetes	440 (19.3%)
Neoadjuvant therapy	137 (6.0%)
Minimally invasive surgery‡	1127 (49.3%)
Operation duration (minutes), median (i.q.r.)	182 (141–241)
No intraoperative drains placed	291 (12.7%)
Estimated blood loss (ml), median (i.q.r.)	200 (50–500)
90-day mortality	34 (1.5%)
Readmission	343 (15.0%)
Length of initial hospital stay (days), median (i.q.r.)	7 (5–12)
Reoperation	138 (6.0%)
POPF, B or C grade§	497 (21.8%)
PPH, B or C grade	108 (4.7%)
DGE, B or C grade	63 (2.8%)
**Tumour histology**	
PDAC	650 (28.5%)
NET	584 (25.6%)
IPMN	296 (13.0%)
Other benign	505 (22.1%)
Other malign	192 (8.4%)
Dysplasia	17 (0.7%)
Non-neoplastic	40 (1.7%)

^*^Values are *n* (%) unless otherwise stated. i.q.r., interquartile range; BMI, body mass index; POPF, postoperative pancreatic fistula; PPH, postpancreatectomy haemorrhage; DGE, delayed gastric emptying; PDAC, pancreatic ductal adenocarcinoma; NET, neuroendocrine tumour; IPMN, intraductal papillary mucinous neoplasm. †History of myocardial infarction, heart failure or coronary artery disease. ‡Laparoscopic or robotic. §ISGPS 2016 classification.

POPF distribution varied between the centres for grade B and C POPF (*P* < 0.001), for grade B POPF only (*P* < 0.001), and for grade C POPF only (*P* < 0.001) (*Fig. S1*). The POPF-related CCI was also statistically significantly different between the nine centres (*Fig. S2*), but in post hoc testing only one centre contributed significantly to this difference by having a lower CCI than others (*Fig. S3*).

### POPF-related interventions

Administration of antibiotics was the most frequent intervention for POPF, with 358 of 497 patients with POPF (72.0%) receiving antibiotics as a treatment, followed by percutaneous intervention (243 of 497 (48.9%)) (*Table 2*). Only 151 of 497 patients with POPF (30.4%) were treated with one intervention modality, whereas most often the number of modalities was two (186 of 497 patients with POPF (37.4%)) (*Table 2*).

**Table 2:** Frequency of different treatment modalities used for postoperative pancreatic fistula in patients undergoing left pancreatectomy

POPF treatment modality	All patients* (*n* = 2284)	Patients with POPF* (*n* = 497)
Prolonged drainage (> 21 days)	205 (9.0%)	205 (41.2%)
Antibiotics	358 (15.7%)	358 (72.0%)
SSA (octreotide)	86 (3.8%)	86 (17.3%)
Percutaneous intervention	243 (10.6%)	243 (48.9%)
**Endoscopic intervention**	92 (4.0%)	92 (18.5%)
ERCP/with pancreatic duct stent	66 (2.9%)	66 (13.3%)
ERCP/without stent	5 (0.2%)	5 (1.0%)
Pseudocystogastrostomy	16 (0.7%)	16 (3.2%)
Data missing	5 (0.2%)	5 (1.0%)
Angiographic intervention	33 (1.4%)	33 (6.6%)
Reoperation (for POPF)	37 (1.6%)	37 (7.4%)
**Number of unique treatment modalities per patient (min 0, max 7)†**		
0	1775 (77.7%)	0 (0%)
1	151 (6.6%)	151 (30.4%)
2	186 (8.1%)	186 (37.4%)
3	116 (5.1%)	116 (23.3%)
4	38 (1.7%)	38 (7.6%)
5 or more	6 (0.3%)	6 (1.2%)
Duration of initial drainage (BL POPF‡) (days), median (i.q.r.)	5 (4–6)	
Duration of initial drainage (B POPF‡)(days), median (i.q.r.)	15 (5–31)	
Duration of initial drainage (C POPF‡)(days), median (i.q.r.)	26 (8–45)	

^*^Values are *n* (%) unless otherwise stated. POPF, postoperative pancreatic fistula; SSA, somatostatin analogue; ERCP, endoscopic retrograde cholangiopancreatography. †Any combination of prolonged drainage, antibiotics, somatostatin analogue, percutaneous, endoscopic or angiographic intervention, or reoperation, each counted once. ‡ISGPS 2016 classification

The most frequent combination of interventions (two or more) for POPF was synchronous administration of antibiotics and percutaneous intervention (79 of 497 (15.9%)), followed by prolonged drainage with antibiotics (40 of 497 (8.0%)), prolonged drainage with antibiotics and percutaneous intervention (31 of 497 (6.2%)), and antibiotics, percutaneous intervention, and administration of somatostatin analogue (16 of 497 (3.2%)).

The reoperation rate in the whole cohort was 6.0% (138 of 2284), and the POPF-related reoperation rate was 1.6% (37 of 2284). The overall postoperative mortality rate was 1.5% (34 of 2284), and the POPF-related mortality rate was 0.6% (14 of 2284).

### POPF-related morbidity

**Fig. 2:**
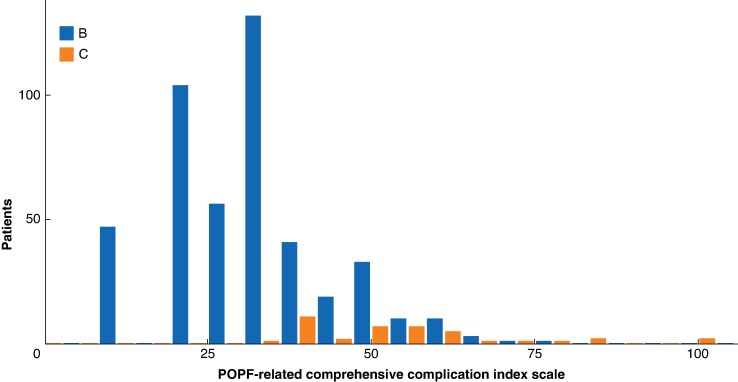
Histogram of postoperative pancreatic fistula-related Comprehensive Complication Index, stratified by ISGPS 2016 POPF grade, in 497 patients who underwent left pancreatectomy and developed postoperative pancreatic fistula

The POPF-related median (i.q.r.) CCI in the whole cohort was 33.5 (22.6–39.7). The median (i.q.r.) CCI was 33.5 (22.6–39.5) for grade B POPF and 50.2 (40.6–58.4) for grade C POPF (*P* < 0.001). However, there was a significant overlap in the CCI distribution between grades B and C POPF, implying that some patients with B POPF experienced higher cumulative morbidity than some patients with C POPF (*Fig. 2*). Using a cutoff of 42.7 CCI (which equals the total cumulative morbidity of one grade IIIa plus one grade IIIb complication) to illustrate this difference: 69 patients with B POPF (15.1%) had a morbidity higher than 42.7 and 12 patients with C POPF (30.0%) had a morbidity lower than 42.7.

### POPF subclassification

The distribution of POPF subclasses is presented in *Fig. 1* and *Table S2*. There was a statistically significant rising trend for readmissions (*P* < 0.001), PPH (*P* = 0.002), length of initial hospital stay (*P* < 0.001), and POPF-related CCI (*P* < 0.001) from B1 to B4 POPF (*Table S1*). Whereas the incidence of reoperations (for any reason) also increased between B1 (1 of 48 (2.1%)), B2 (5 of 135 (3.7%)), B3 (13 of 175 (7.4%)), and B4 (11 of 99 (11.1%)), this was not statistically significant (*P* = 0.074). Postoperative mortality was similar throughout the B subclasses (0–3.7–1.1–2.0%; *P* = 0.30), but significantly higher for grade C POPF (12.5%; *P* = 0.002).

**Fig. 3:**
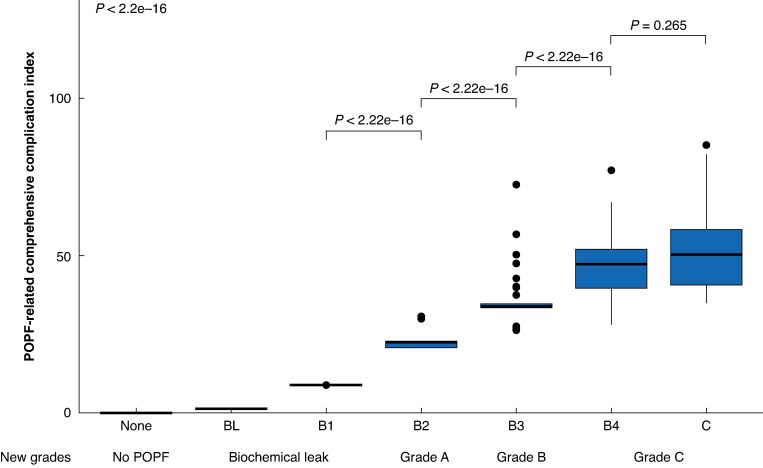
Boxplots of postoperative pancreatic fistula-related Comprehensive Complication Index in ISGPS 2016 classification (including B subclasses) and in all proposed new POPF grades in 2284 patients who underwent left pancreatectomy Kruskal–Wallis; *P* < 2.2e-16.

The CCI ranged between 8.7 and 58.3 for the B subclasses, and between 34.8 and 100 (85.2 when excluding the two patients that died) for grade C POPF (*Fig. 3*). In post hoc pairwise analyses, the median CCI was similar between grade B4 and C POPF (*P* = 0.265).

### New proposed POPF grading system

The refined POPF grading system consists of grades A, B, and C (*Table 3* and *Fig. 1*). As B1 had outcomes comparable to biochemical leak and no POPF (*Table S1*), prolonged drainage only was regarded as a clinically insignificant alteration and does not lead to clinically relevant POPF diagnosis. B2 (pharmacological intervention only) was associated with an increased readmission rate and CCI and is thus regarded as a new grade A POPF. B3 (percutaneous intervention) was associated with a growing readmission rate and CCI, but with a LOS similar to B2, and is regarded as a new grade B POPF. B4 (endoscopic or angiographic intervention) and C demonstrated readmission rates similar to B3, but a significantly longer LOS and increased reoperation (any reason) rate. B4 and C also demonstrated a similar CCI and are thus combined as the new grade C POPF in the proposed reclassification.

**Table 3:** Incidence of different postoperative outcomes in the proposed new classification for postoperative pancreatic fistula after left pancreatectomy

		No POPF (*n* = 1338)	Biochemical leak (new) (*n* = 497)	A POPF (new) (*n* = 135)	B POPF (new) (*n* = 175)	C POPF (new) (*n* = 139)	*P* *	Effect size (per group)† OR (95% c.i.) for categorical variables, beta coefficient (95% c.i.) for continuous variables
Readmission		109 (8.1%)	57 (11.5%)	37 (27.4%)	82 (46.9%)	58 (41.7%)	< 0.001	2.21 (1.98–2.46)
Postpancreatectomy haemorrhage (grade B and C)		47 (3.5%)	13 (2.6%)	5 (3.7%)	10 (5.7%)	34 (24.5%)	< 0.001	1.98 (1.70–2.32)
Delayed gastric emptying (grade B and C)		32 (2.4%)	11 (2.2%)	1 (0.7%)	8 (4.6%)	11 (7.9%)	< 0.001	1.49 (1.20–1.85)
Reoperation (for any reason)		63 (4.7%)	10 (2.0%)	5 (3.7%)	13 (7.4%)	47 (33.8%)	< 0.001	2.14 (1.86–2.47)
Severe morbidity (Clavien–Dindo IIIa or worse)		158 (11.8%)	62 (12.5%)	26 (19.3%)	175 (100%)	139 (100%)	< 0.001	8.43 (6.88–10.33)
90-day mortality		15 (1.1%)	5 (1.0%)	4 (3.0%)	2 (1.1%)	8 (5.8%)	< 0.001	1.64 (1.24–2.17)
Length of initial hospital stay (days), median (i.q.r)		6 (5–9)	8 (6–11)	10 (7–15)	11 (7–18)	20 (8–32)	< 0.001	+4.7 (4.1–5.3)
POPF-related Comprehensive Complication Index (0–100)‡, median (i.q.r)		0	0	22.6 (20.9–22.6)	33.5 (33.5–34.6)	43.6 (39.7–48.3)	< 0.001	+10.4 (9.8–10.9)
Distribution of Clavien–Dindo grade (up to 30th POD)	0	754 (56.6%)	226 (45.7%)	0	0	0	< 0.001	
	I	175 (13.1%)	64 (13.0%)	0	0	0		
	II	246 (18.5%)	144 (29.1%)	109 (80.7%)	0	0		
	IIIa	85 (6.4%)	42 (8.5%)	20 (14.8%)	167 (95.4%)	22 (15.8%)		
	IIIb	47 (3.5%)	11 (2.2%)	1 (0.7%)	5 (2.9%)	93 (66.9%)		
	IVa	14 (1.1%)	3 (0.6%)	2 (1.5%)	1 (0.6%)	11 (7.9%)		
	IVb	1 (0.1%)	1 (0.2%)	1 (0.7%)	2 (1.1%)	6 (4.3%)		
	V	10 (0.8%)	3 (0.6%)	2 (1.5%)	0	7 (5.0%)		

^*^Values are *n* (%) unless otherwise stated. POPF, postoperative pancreatic fistula; OR, odds ratio; c.i., confidence interval. *Kruskal–Wallis test for continuous variables and χ^2^ test for categorical variables. †Effect size calculated with logistic regression for categorical variables and linear regression for continuous variables. Effect size is reported per increase of POPF grade. No POPF and biochemical leak are pooled in this analysis. ‡Effect size for Comprehensive Complication Index increase is reported only for grades A, B, and C POPF because biochemical leak by definition has a 0 POPF-related Comprehensive Complication Index.

*Figures S4* and *S5* showed the centre-wise variation in the CCI between the POPF grades for both the new proposed POPF classification system (*Fig. S4*) and the ISGPS POPF classification (*Fig. S5*). The new classification seems to better discriminate between low (grade A), intermediate (grade B), and high (grade C) POPF-related morbidity, without much overlap between grades between different centres. Grade-wise variation in the CCI is also smaller in the new system, possibly indicating more comparable POPF classification between different centres. The CCI i.q.r for the ISGPS grades B POPF and C POPF varies between 11.4 and 12.0, indicating a large variation. For the new POPF classification, the i.q.r. for A, B, and C POPF varies between 1.1 and 8.6, indicating a lower variation within grades.

## Discussion

In this study with 2284 patients, 497 experienced ISGPS 2016 grade B or C POPF. The total cumulative morbidity and other short-term outcomes experienced by patients were highly variable both inside ISGPS 2016 B and C POPF, and between participating centres. It was observed that the current definition, especially for grade B POPF, included a highly heterogenous population; some patients with B POPF experienced negligible cumulative morbidity and rarely other sequelae, whereas others experienced prolonged and complicated postoperative courses on par with C POPF. A refined grading system for POPF after left pancreatectomy, consisting of three grades (A, B, and C) with relevant, systematically worsening outcomes was proposed.

When analysing the different categories inside ISGPS 2016 B POPF, a significant rising trend in morbidity was noted. Patients with B1 POPF (prolonged drainage) demonstrated postoperative courses similar to patients without any signs of POPF and patients with biochemical leak. When moving further to subclasses B2 (pharmacological intervention) and B3 (percutaneous intervention) POPF, a rising trend in readmissions, other pancreatectomy-specific complications, and a POPF-related CCI were observed. Subclass B4 POPF on the other hand showed cumulative morbidity and length of initial hospital stay comparable to C POPF. C POPF was associated with significantly higher postoperative mortality and reoperation rates compared with B4 POPF, but this was expected due to the fact that the C POPF definition is partly based on these outcomes occurring.

Dropping prolonged drainage from POPF severity classification seems justifiable, especially given the recent results of the PANDORINA trial^15^, as routine intra-abdominal drainage is not recommended after left pancreatectomy. With this shift to an era of drainless left pancreatectomy, routine monitoring for biochemical leaks, the common nominator for current POPF definition, will likely become obsolete. However, amylase-rich intra-abdominal fluid will still likely remain the standard for POPF diagnosis in refined grades B and C (where sampling is feasible), whereas refined grade A (pharmacological treatment without sampling) might not require evidence of elevated amylase levels in the fluid collection.

In this study, a refined and more clinically relevant grading for POPF after left pancreatectomy is proposed. Whereas biochemical leak grade will probably become obsolete due to the aforementioned reasons, it is still differentiated from no POPF and the first clinically relevant grade A POPF in the refined system, similar to the ISGPS definition. Biochemical leaks now include patients with elevated drain fluid amylase and/or prolonged routine drainage with no other clinical consequences. This grade demonstrated outcomes similar to the no POPF grade. Grade A consists of patients with POPFs that are limited to pharmacological treatment only. These patients rarely experience other POPF-related complications like DGE or PPH, but their readmission rate and LOS are markedly increased from no POPF (also including biochemical leak). Grade B requires percutaneous intervention and consists of patients with progressively worsening outcomes, especially higher readmission rates and CCI, but also slightly higher rates of reoperation (for other reasons than POPF). The new grade C now encompasses a larger group of patients that undergo endoscopic, angiographic, or surgical reintervention. Undergoing endoscopic or angiographic procedures require special intervention room resources and general anaesthesia, or at least readiness for it. Differences in regional or institutional protocols, expertise, and availability of course affect the threshold at which an endoscopic intervention for example is initiated. Some centres might opt for endoscopic interventions earlier, or instead of percutaneous interventions, than others^16^. Undergoing these procedures due to POPF clearly shifts its burden to a higher level. Patients with new grade C POPF experience significantly more PPH, DGE, and reoperation compared with those with A and B POPF. In this new system, all grades from A to C can be regarded as clinically relevant and thus the term ‘clinically relevant’ becomes redundant.

When compared with ISGPS B and C POPF, the new classification demonstrated much smaller variation in the CCI inside the grades, perhaps offering a more comparable grading system between different centres. The purpose of the proposed refined POPF grading system is not to disrupt established reporting frameworks currently in use, namely the 2016 ISGPS classification.

There are some major limitations with this study. The data were collected retrospectively and are thus prone to misclassification errors, and there were some major differences between centres in terms of perioperative protocols, that is, routine or selective drainage, and use of somatostatin analogues or not, and so on, which might affect how ‘easily’ POPF is diagnosed or a ‘clinically relevant change’ in postoperative management is met. Data collection was also performed by each participating centre, which introduces some heterogeneity into the data. These can be partly reflected in the varying POPF incidence and overall POPF-related morbidity between the participating centres. Variation in POPF incidence is inevitable due to different perioperative protocols and hospital volumes. Having a classification system with small in-grade variation between different centres regardless of POPF incidence is more important. Patients undergoing multivisceral resections (other than splenectomy) were not included, potentially limiting the generalizability of the present results to this patient group. The ISGPS definition has been used for pancreatic surgery with multivisceral resections, and the present refined classification system used the same definition.

Another limitation is that the CCI was calculated only for patients with POPF and only using POPF-related interventions/treatments. Thus, comparison of the total cumulative morbidity of patients with POPF with patients without POPF, or consideration of postoperative morbidity due to other complications (such as pneumonia or cardiovascular complications) is not possible. Whereas this slightly out-of-the-box use of the CCI might underestimate the total morbidity of patients with numerous other postoperative complications, it still aligns with the main aim of this study, which was to assess variation in morbidity due to interventions aimed at POPF. Incorporating quality-of-life measurements or other patient-reported outcomes would have been an important addition to the present analysis, but unfortunately, due to the study design, this was not feasible. The recently identified challenge with current POPF definition interobserver variability was also present in the present study. This was mitigated in part by systematically collecting all interventions aimed at POPF, and grading POPF based on these. The authors believe the current ISGPS 2016 definition to be adequately sensitive in identifying patients with POPF, but lacking more in specificity, thus warranting this reappraisal.

This study used the CCI to capture the cumulative extent of POPF-related morbidity^14^. One question arising from this study is whether redefining POPF categories or its definitions is important, or whether the POPF-related CCI as a primary outcome should be used in future studies. As a continuous variable, it contains more information and would allow for a more precise comparison of outcomes. Clinical relevance and associated morbidity of different POPF grades might differ greatly between left pancreatectomy and pancreatoduodenectomy as the operations are very different. No pancreatoenteric anastomosis is performed in left pancreatectomy, which is associated with less bacterial growth in POPF collections^17^. Assessing POPF-related morbidity and reappraising its classification in left pancreatectomy is very important to ensure correct endpoints are used in future clinical trials.

Moving forward, the refined POPF classification should enhance both research and quality monitoring by providing a reference for more standardised and clinically meaningful assessment of postoperative outcomes. By reducing heterogeneity within POPF grades, this system should improve comparability across studies and facilitate more accurate benchmarking in surgical registries. Its adoption in quality monitoring initiatives could help reduce interobserver variability, optimize institutional performance assessments, and should guide refinements in perioperative care protocols. Prospective validation and international consensus will be essential next steps to ensure widespread applicability.

## Supplementary Material

zraf149_Supplementary_Data

## Data Availability

Due to the international multicentre setting of this study and varying regional and national legislations, no data are available for sharing.
