# Supplementary material for: Classification of postoperative pancreatic fistula after left pancreatectomy: international multicentre cohort study
Source: BJS Open. 2025 Dec 3;9(6):zraf149. doi: 10.1093/bjsopen/zraf149 (PMC12673845; doi:10.1093/bjsopen/zraf149)
Supplement: zraf149_Supplementary_Data [file zraf149_supplementary_data.docx]

**Classification of postoperative pancreatic fistula after left pancreatectomy: international multicenter cohort study**

Akseli Bonsdorff MD PhD^1^, William Yu MD^2^, Jakob Kirkegård MD PhD^3^, Charles de Ponthaud MD^4^, Trond Kjeseth MD^5,6^, Poya Ghorbani MD PhD^2^, Johanna Wennerblom MD PhD^7^, Caroline Williamson MD PhD^8^, Alexandra W Acher MD^9^, Manoj Thillai MD^10^, Timo Tarvainen MD^1^, Aki Uutela MD PhD^10,16^, Jukka Sirén MD PhD^1^, Arto Kokkola MD PhD^1^, Dyre Kleive MD PhD^5^, Mushegh Sahakyan MD PhD^11,12,13^, Rolf E Hagen MD^14^, Andrea Lund MD^3^, Mette Fugleberg Nielsen MD^3^, Richard Fristedt MD PhD^8^, Christina Biörserud PhD^7^, Svein Olav Bratlie MD PhD^7^, Bobby Tingstedt MD PhD^8^, Knut J. Labori MD PhD^5,15^, Sébastien Gaujoux MD PhD^4^, Stephen J Wigmore MD FRCSED^10^, Julie Hallet MD^9^, Ernesto Sparrelid MD PhD^2^, Ville Sallinen MD PhD^1,16^

^1 Helsinki University Hospital and University of Helsinki, Department of Gastroenterological Surgery, Helsinki, Finland^

^2 Division of Surgery and Oncology, Department of Clinical Science, Intervention, and Technology, Karolinska Institutet, Karolinska University Hospital, Stockholm, Sweden^

^3 Aarhus University Hospital, Department of Surgery, HPB section and Institute for Clinical Medicine, Aarhus University, Aarhus, Denmark^

^4 Department of Digestive, Hepato-Pancreato-Biliary Surgery and Liver Transplantation, AP-HP Pitié-Salpêtrière Hospital, Sorbonne Université Paris, France^

^5 Department of Hepato-Pancreato-Biliary Surgery, Rikshospitalet, Oslo University Hospital, Oslo, Norway^

^6 Department of Clinical Medicine, University of Bergen, Bergen, Norway^

^7 Department of Surgery, Sahlgrenska University Hospital, Gothenburg, Sweden^

^8 Lund University, Department of Surgery, Skåne University Hospital at Lund, Lund, Sweden^

^9 Department of Surgery, University of Toronto and Sunnybrook Health Sciences Centre, Toronto, Ontario, Canada^

^10 Hepatobiliary and Pancreatic Unit & Edinburgh Transplant Unit, University of Edinburgh, Royal Infirmary, EH16 4SA, Edinburgh, UK^

^11 Department of Surgery, Vestre Viken Hospital Trust, Ringerike Hospital, Hønefoss, Norway^

^12 The Intervention Centre, Oslo University Hospital, Rikshospitalet, Oslo, Norway^

^13 Department of Surgery N1, Yerevan State Medical University, Yerevan, Armenia^

^14 Department of Surgery, Vestfold Hospital Trust, Tønsberg Norway^

^15 Institute of Clinical Medicine, University of Oslo, Oslo, Norway^

^16 Helsinki University Hospital and University of Helsinki, Department of Transplantation and Liver Surgery, Helsinki, Finland^

**Corresponding author.** Ville Sallinen, Adjunct professor, Head of Department, MD, PhD, Helsinki University Hospital and University of Helsinki, [ville.sallinen@helsinki.fi](mailto:ville.sallinen@helsinki.fi)

Haartmaninkatu 4, 00029 Helsinki, Finland. **ORCID ID**; 0000-0001-5394-4169, **Twitter**; @villesallinen

**Supplementary Materials - Index**

| **Supplementary Figures and Tables** |  |
| --- | --- |
| **Supplementary table 1** | *page 3* |
| **Supplementary table 2** | *page 4* |
| **Supplementary figure 1** | *page 5* |
| **Supplementary figure 2** | *page 6* |
| **Supplementary figure 3** | *page 7* |
| **Supplementary figure 4** | *page 8* |
| **Supplementary figure 5** | *page 9* |

**Supplementary table 1.** Perioperative factors of 2284 patients undergoing left pancreatectomy, reported per participating center.

Abbreviations: BMI; body mass index, IQR; inter-quartile range, PDAC; pancreatic ductal adenocarcinoma, IPMN; intraductal papillary mucinous neoplasm, NET; neuroendocrine tumor, ISGPS; international study group for pancreatic surgery, POPF; postoperative pancreatic fistula.

|  | | | **Helsinki** | **Stockholm** | **Aarhus** | **Oslo** | **Paris** | **Lund** | **Gothenburg** | **Edinburgh** | **Toronto** |
| --- | --- | --- | --- | --- | --- | --- | --- | --- | --- | --- | --- |
| **Sample size** | | | *266 (11.6%)* | *402 (17.6%)* | *199 (8.7%)* | *538 (23.6%)* | *173 (7.6%)* | *170 (7.4%)* | *200 (8.8%)* | *151 (6.6%)* | *185 (8.1%)* |
| **Age, median (IQR)** | | | 65 (54-71) | 67 (56-73) | 63 (51-70) | 65 (54-72) | 63 (50-72) | 68 (56-74) | 69 (55-75) | 61 (48-69) | 64 (54-71) |
| **Male sex, n (%)** | | | 107 (40.2%) | 183 (45.5%) | 96 (48.2%) | 281 (52.2%) | 91 (52.6%) | 66 (38.8%) | 86 (43.0%) | 62 (41.1%) | 86 (46.5%) |
| **BMI, median (IQR)** | | | 26.2 (23.8-29.8) | 25.8 (22.7-29.3) | 26.0 (22.9-29.4) | 25.5 (22.8-28.6) | 25.0 (22.0-28.0) | 25.3 (22.8-28.0) | 27.0 (23.0-30.0) | 26.3 (22.6-31.0) | 27.0 (23.2-30.9) |
| **Operation type** | | Open | 118 (44.4%) | 337 (83.8%) | 176 (88.4%) | 38 (7.1%) | 110 (63.6%) | 102 (60.0%) | 72 (36.0%) | 111 (73.0%) | 91 (49.2%) |
|  |  | Lap | 113 (42.5% | 43 (10.7%) | 23 (11.5%) | 500 (93.1%) | 62 (35.6%) | 61 (35.9%) | 110 (55.0%) | 41 (27.0%) | 93 (50.3%) |
|  |  | Rob | 34 (12.8%) | 22 (5.5%) | 0 | 0 | 1 (0.6%) | 7 (4.1%) | 17 (8.5%) | 0 | 0 |
| **Transection method** | | Stapler | 226 (85.0%) | 330 (82.1%) | 188 (94.0%) | 537 (99.8%) | 96 (55.5%) | 119 (70.0%) | 194 (97.0%) | 125 (82.8%) | 165 (89.2%) |
|  |  | Hand-sewn | 40 (15.0%) | 71 (17.7%) | 3 (1.5%) | 0 | 74 (42.8%) | 51 (30.0%) | 3 (1.5%) | 26 (17.2%) | 0 |
|  |  | Other | 0 | 0 | 6 (3.0%) | 1 (0.2%) | 0 | 0 | 3 (1.5%) | 0 | 20 (10.8%) |
| **Use of somatostatin analogue** | | None | 123 (46.2%) | 293 (72.9%) | 198 (99.5%) | 530 (98.5%) | 107 (61.8%) | 6 (3.5%) | 192 (96.0%) | 142 (94.0%) | 176 (95.1%) |
|  |  | Prophylactic | 143 (53.8%) | 0 | 0 | 0 | 54 (31.2%) | 160 (94.1%) | 7 (3.5%) | 5 (3.4%) | 3 (1.6%) |
|  |  | Treatment | 0 | 109 (27.1%) | 1 (0.5%) | 8 (1.5%) | 12 (6.9%) | 4 (2.4%) | 1 (0.5%) | 0 | 6 (3.2%) |
| **Arterial resection** | | | 6 (2.2%) | 4 (1.0%) | 0 | 2 (0.4%) | 1 (0.6%) | 2 (1.2%) | 4 (2.0%) | 3 (2.0%) | 2 (2.3%) |
| **Venous resection** | | | 23 (8.6%) | 13 (3.3%) | 0 | 15 (3.8%) | 8 (4.6%) | 6 (3.5%) | 28 (14.0%) | 0 | 9 (10.5%) |
| **Perioperative intra-abdominal drainage** | | | 266 (100%) | 402 (100%) | 44 (22.1%) | 538 (100%) | 170 (98.3%) | 142 (83.5%) | 199 (99.5%) | 138 (91.4%) | 92 (49.7%) |
| **Tumor histology / pathologist’s report** | | PDAC | 69 (25.9%) | 104 (25.9%) | 59 (29.6%) | 146 (27.1%) | 70 (40.5%) | 61 (35.9%) | 55 (27.5%) | 26 (17.2%) | 60 (32.4%) |
|  |  | IPMN | 28 (10.5%) | 97 (24.1%) | 6 (3.0%) | 53 (9.9%) | 15 (8.7%) | 37 (21.8%) | 31 (15.5%) | 16 (10.6%) | 13 (7.0%) |
|  |  | NET | 78 (29.3%) | 75 (18.7%) | 66 (33.2%) | 144 (26.8%) | 41 (23.7%) | 27 (15.9%) | 59 (29.5%) | 41 (27.2%) | 53 (28.6%) |
|  |  | Other malign | 22 (8.3%) | 31 (7.7%) | 16 (8.0%) | 45 (8.4%) | 15 (8.7%) | 14 (8.2%) | 7 (3.5%) | 27 (17.9%) | 15 (8.1%) |
|  |  | Other benign | 56 (21.1%) | 95 (23.6%) | 40 (20.1%) | 140 (26.0%) | 32 (18.5%) | 30 (17.6%) | 43 (21.5%) | 31 (20.5%) | 38 (20.5%) |
|  |  | Dysplasia | 6 (2.3%) | 0 | 0 | 1 (0.2%) | 0 | 1 (0.6%) | 3 (1.5%) | 1 (0.7%) | 4 (2.2%) |
|  |  | Not diagnostic | 2 (0.8%) | 0 | 12 (6.0%) | 9 (1.7%) | 0 | 0 | 2 (1.0%) | 9 (6.0%) | 2 (1.1%) |
| **ISGPS POPF grade B or C, n(%)** | | | 54 (20.3%) | 158 (39.3%) | 16 (8.0%) | 112 (20.8%) | 64 (37.0%) | 21 (12.4%) | 18 (9.0%) | 14 (9.3%) | 40 (21.6%) |
| **Refined POPF, n(%)** | **A** | | 16 (6.0%) | 71 (17.8%) | 3 (1.5%) | 13 (2.4%) | 12 (6.9%) | 7 (4.1%) | 0 | 1 (0.7%) | 12 (6.5%) |
|  | **B** | | 17 (6.4%) | 51 (12.8%) | 7 (3.5%) | 52 (9.7%) | 3 (1.7%) | 12 (7.1%) | 5 (2.5%) | 8 (5.3%) | 20 (10.8%) |
|  | **C** | | 20 (7.5%) | 27 (6.8%) | 6 (3.0%) | 38 (7.1%) | 36 (20.8%) | 0 | 2 (1.0%) | 6 (3.9%) | 4 (2.2%) |

**Supplemental Table 2.** Incidence of different postoperative outcomes in all different subclasses of postoperative pancreatic fistula in patients undergoing left pancreatectomy.

| **Outcome / POPF grade** | **None (n=1338)** | **BL**  **(n=449)** | **B1 (n=48)** | **B2 (n=135)** | **B3 (n=175)** | **B4 (n=99)** | **C (n=40)** | ***P***  ***BL vs. B1*** | ***P***  ***B1 vs. B2*** | ***P***  **B2 vs. B3** | ***P***  ***B3***  **vs.**  **B4** | ***P***  **B4**  **vs.**  **C** |
| --- | --- | --- | --- | --- | --- | --- | --- | --- | --- | --- | --- | --- |
| **Readmission** | 109 (8.4%) | 50 (11.3%) | 7 (14.6%) | 37 (28.7%) | 82 (47.1%) | 41 (41.4%) | 17 (43.6%) | 0.656 | 0.048 | 0.002 | 0.432 | 0.967 |
| **Postpancreatectomy hemorrhage (grade B and C)** | 47 (3.5%) | 13 (2.9%) | 0 | 5 (3.8%) | 10 (5.7%) | 14 (14.1%) | 19 (47.5%) | 0.472 | 0.396 | 0.601 | 0.032 | <0.001 |
| **Delayed gastric emptying (grade B and C)** | 32 (2.4%) | 11 (2.4%) | 0 | 1 (0.7%) | 8 (4.6%) | 6 (6.1%) | 5 (12.5%) | 0.562 | 0.550 | 0.046 | 0.591 | 0.354 |
| **Reoperation (for any reason)** | 63 (4.7%) | 9 (2.0%) | 1 (2.1%) | 5 (3.7%) | 13 (7.4%) | 11 (11.1%) | 36 (90.0%) | 0.970 | 0.588 | 0.252 | 0.416 | <0.001 |
| **90-day mortality** | 15 (1.1%) | 5 (1.1%) | 0 | 5 (3.7%) | 2 (1.1%) | 2 (2.0%) | 5 (12.5%) | 0.462 | 0.403 | 0.263 | 0.954 | 0.033 |
| **Duration of initial drainage** | 0 (0-5) | 5 (4-6) | 34 (24-48) | 15 (8-30) | 8 (4-23) | 13 (4-30) | 26 (8-45) | <0.001 | <0.001 | <0.001 | 0.178 | 0.057 |
| **Length of initial hospital stay (d)** | 6 (5-9) | 7 (6-11) | 13 (7-19) | 10 (7-15) | 11 (7-18) | 20 (8-29) | 21 (11-49) | <0.001 | 0.332 | 0.291 | <0.001 | 0.162 |
| **POPF related Comprehensive Complication Index (0-100)** | 0 | 0 | 8.7 (8.7-8.7) | 22.6 (20.9-22.6) | 33.5 (33.5-34.6) | 47.4 (39.7-52.1) | 50.2 (40.6-58.4) |  | <0.001 | <0.001 | <0.001 | 0.265 |

Abbreviations: POPF; postoperative pancreatic fistula, BL; biochemical leak.

**Supplemental Figure 1.** Distribution of postoperative pancreatic fistula grade (ISGPS 2016) in all the nine participating centers.

**
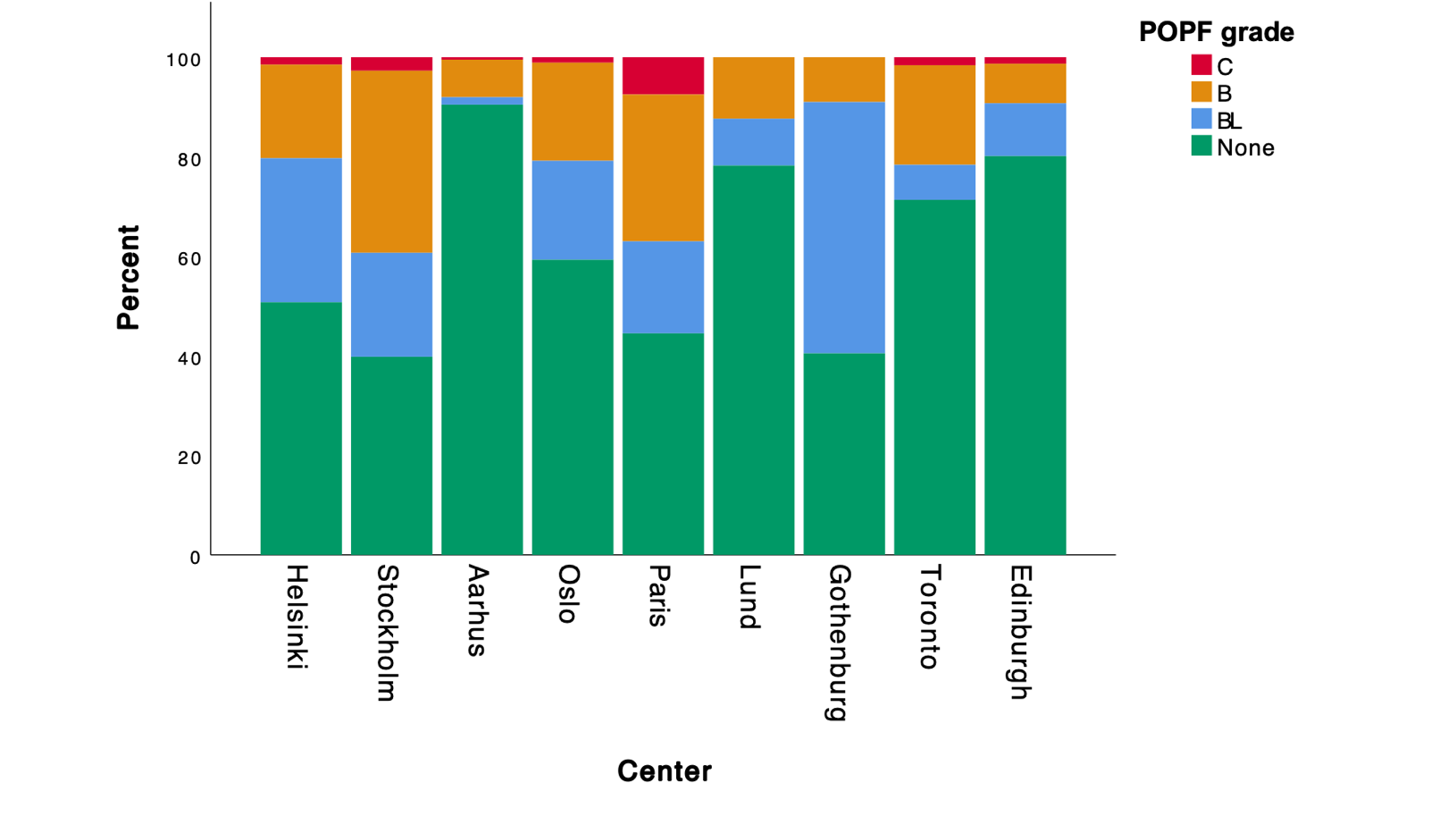
**

**Supplemental Figure 2.** Distribution of postoperative pancreatic fistula related Comprehensive Complication Index of 2284 patients undergoing left pancreatectomy at nine different high-volume pancreatic surgery centers.

**
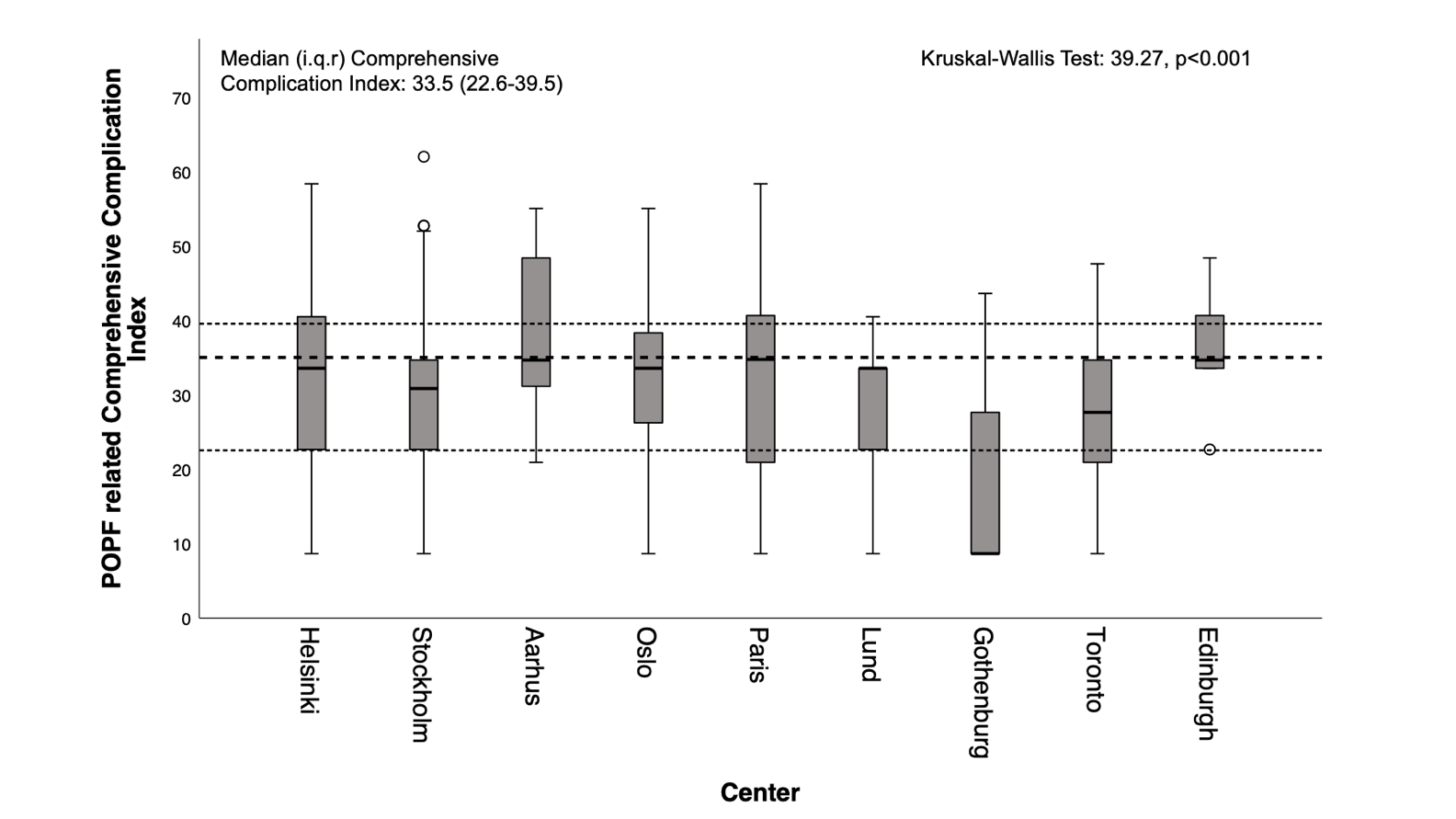
**

**Footnote:** Dashed lines represent population median (i.q.r.) pancreatic fistula related Comprehensive Complication Index.

**Supplemental Figure 3.** Pairwise comparison of postoperative pancreatic fistula related Comprehensive Complication Index between nine different high-volume pancreatic surgery centers.

**Supplemental Figure 4.** Center wise distribution of CCI, stratified by the proposed new POPF classification system. Dashed horizontal lines represent median CCI for the corresponding POPF grade.

**
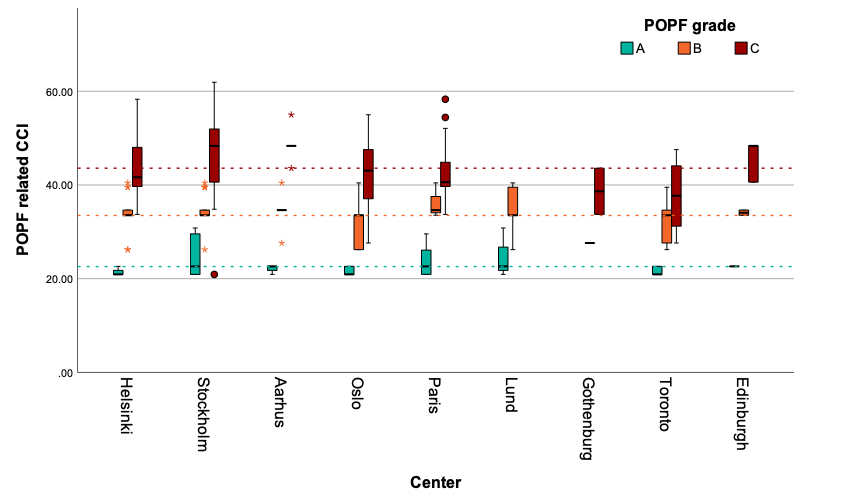
**

**Footnote:** line splitting the box is median, box edges represent quartiles, whiskers represent 95% confidence interval and points represent outliers.

**Supplemental Figure 5.** Center wise distribution of CCI, stratified by the ISGPS 2016 POPF classification. Dashed horizontal lines represent median CCI for the corresponding POPF grade.

**
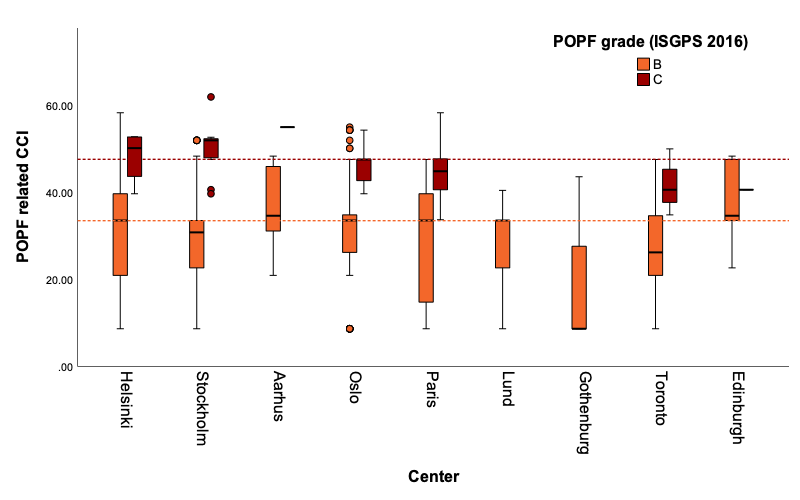
**

**Footnote:** line splitting the box is median, box edges represent quartiles, whiskers represent 95% confidence interval and points represent outliers.
